# Tracing the birth and intrinsic disorder of loops and domains in protein evolution

**DOI:** 10.1007/s12551-024-01251-0

**Published:** 2024-11-20

**Authors:** Gustavo Caetano-Anollés, Fizza Mughal, M. Fayez Aziz, Kelsey Caetano-Anollés

**Affiliations:** 1 Evolutionary Bioinformatics Laboratory, Department of Crop Sciences and Carl R. Woese Institute for Genomic Biology, University of Illinois at Urbana-Champaign, Urbana, IL 61801 USA; 2 Callout Biotech, Albuquerque, NM 87112 USA

**Keywords:** Accretion, Folds, Intrinsic disorder, Molecular evolution, Origin of life, Phylogenomic reconstruction, Protein structure

## Abstract

Protein loops and structural domains are building blocks of molecular structure. They hold evolutionary memory and are largely responsible for the many functions and processes that drive the living world. Here, we briefly review two decades of phylogenomic data-driven research focusing on the emergence and evolution of these elemental architects of protein structure. Phylogenetic trees of domains reconstructed from the proteomes of organisms belonging to all three superkingdoms and viruses were used to build chronological timelines describing the origin of each domain and its embedded loops at different levels of structural abstraction. These timelines consistently recovered six distinct evolutionary phases and a most parsimonious evolutionary progression of cellular life. The timelines also traced the birth of domain structures from loops, which allowed to model their growth ab initio with AlphaFold2. Accretion decreased the disorder of the growing molecules, suggesting disorder is molecular size-dependent. A phylogenomic survey of disorder revealed that loops and domains evolved differently. Loops were highly disordered, disorder increased early in evolution, and ordered and moderate disordered structures were derived. Gradual replacement of loops with α-helix and β-strand bracing structures over time paved the way for the dominance of more disordered loop types. In contrast, ancient domains were ordered, with disorder evolving as a benefit acquired later in evolution. These evolutionary patterns explain inverse correlations between disorder and sequence length of loops and domains. Our findings provide a deep evolutionary view of the link between structure, disorder, flexibility, and function.

## Introduction

**Fig. 1:**
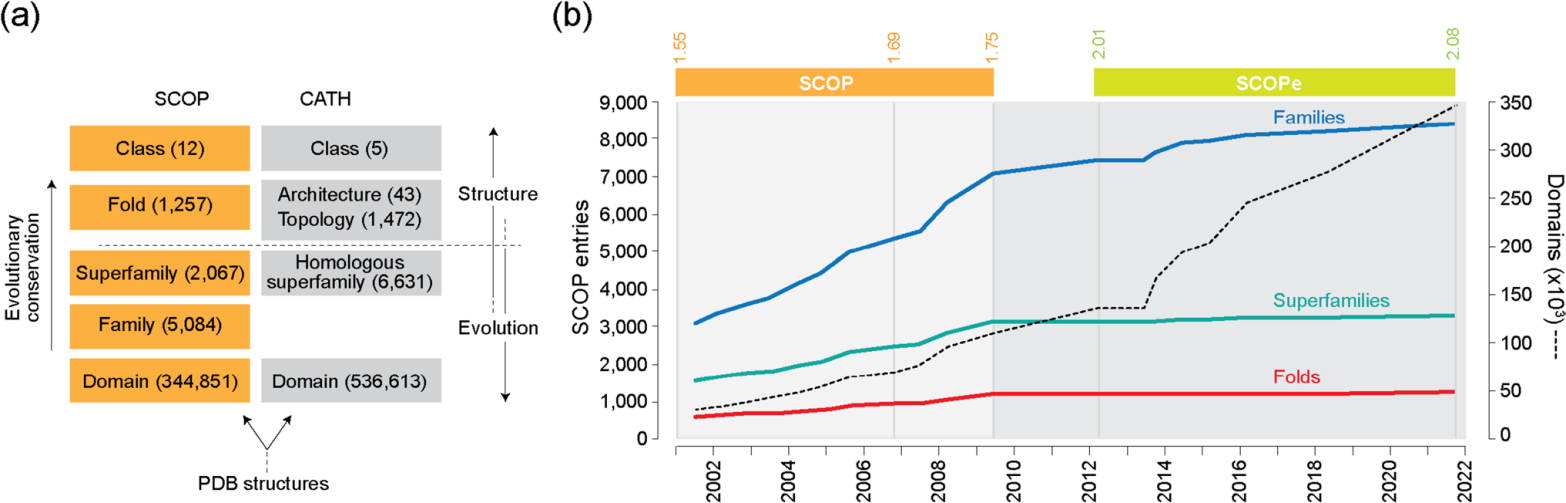
Exploring the complexity of the world of globular proteins through the lens of the structural domain. **a** SCOP and CATH group structural domains based on considerations of structure and evolution. Numbers of taxonomic entries (October 1, 2024) are given in parentheses. **b** Growth of the SCOP and SCOPe hierarchies showing the onset of a plateau of taxonomic growth (dark-shaded section of the timeline)

Proteins are the workhorses of cells and viral life cycles. Their makeup is complex and hierarchical and their evolution entangled (Caetano-Anollés et al. 2009; 2021). Polypeptide chains are structured but also intrinsically flexible. They fold cooperatively into atomic three-dimensional conformations that are compact but that express many layers of molecular organization. Secondary structures, such as α-helices, turns and bends, form asynchronously through “foldon” intermediates (Englander and Mayne 2014) at timescales that vary from microseconds to hours, some facilitated by co-translational chaperoned stabilization along the ribosomal exit tunnel (Holtkamp et al. 2015; Shieh et al. 2015). Long-distance interactions forming strands, bridges, and sheets guide the free-energy landscape stepwise towards the native state (Thommen et al. 2017), avoiding misfolding and facilitating co-translational protein complex assembly pathways (Venezian et al. 2024). These processes are evolutionarily tailored to produce an unprecedented but finite diversity of structural forms. Structural databases, such as SCOP (Murzin et al. 1995), its extended backward-compatible version SCOPe (Fox et al. 2014), and CATH (Orengo et al. 1997), all considered gold standards, use hierarchical classification schemes to catalogue the structural world of proteins. Their units of classification are the *structural domains*, the structural, functional, and evolutionary building blocks of the protein world (Fig. 1a). Climbing up the hierarchies increases conservation and provides a deeper and stable evolutionary view. In fact, newly discovered taxa (e.g., SCOP families, superfamilies, and folds; Fig. 1b) are reaching a plateau, suggesting classification is essentially complete at higher ranks. The existence of a finite but diversifying world of protein structure facilitates an exploration of its origin and evolution. The conserved structural cores of domains rarely evolve by convergent evolution (Gough 2005). However, domains combine in evolution to form a multiplicity of multidomain proteins (Wang and Caetano-Anollés 2009). This generates a network of protein co-options, which can be traced along the 3.8 billion-year (Gy) history of the protein world with time-dependent networks (Aziz and Caetano-Anollés 2021). Protein history is therefore driven by complicated recruitment processes that make evolution reticulated.

Here, we review efforts to understand the origin and evolution of protein domains, the process of accretion responsible for their complexity, and the role that order and disorder (i.e., the sampling of backbone conformations by amino acid residues) have in defining protein structure. We first review our phylogenomic exploration efforts and the methodology used to trace deep protein evolution. Secondly, we turn to “prior molecular states” and describe how they help illuminate the evolutionary origins of protein structure. Lastly, we focus on the evolution of intrinsic disorder and its role in protein flexibility and function.

## Phylogenomic exploration of the protein world

**Fig. 2:**
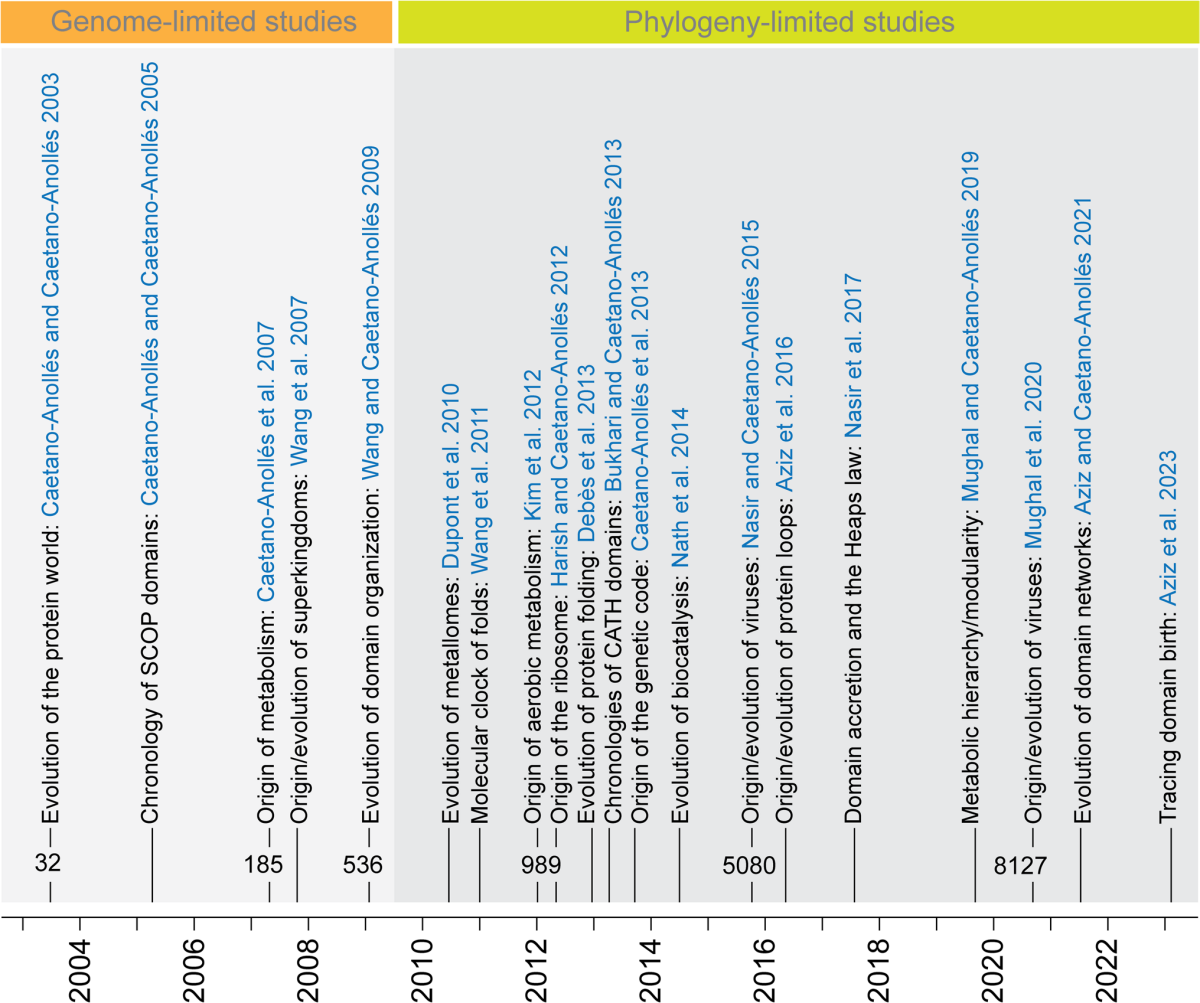
Publication timestamps highlighting 20 out of about 100 phylogenomic studies of proteome evolution at the structural domain level. Numbers of proteomes that were examined are indicated for selected studies. Different gray-scale sections indicate studies being limited by the availability of genomes or the difficulties of phylogenomic reconstruction

Phylogenomic methods can provide global evolutionary descriptions of the protein world (reviewed in Caetano-Anollés et al. 2021). They have been used not only to build chronologies describing the “time of origin” (age) of individual domain structures, but also time-dependent networks describing recruitment processes responsible for both the birth of domains and the generation of multidomain proteins. These efforts began with the advent of the genomic revolution through a phylogenomic analysis of 32 proteomes available at the time (Caetano-Anollés and Caetano-Anollés 2003). Figure 2 shows a timeline of phylogenomic studies conducted in our laboratory. Initial studies were limited by the acquisition of complete and permanent drafts of genomes. With time, however, that limitation was replaced by a focus on solving difficulties of phylogenetic reconstruction, including the problems of large trees, big data, taxon/character sampling, and the effect of organismal lifestyles. Studies tackled the grand challenge of understanding the origin, evolution, and complexity of cellular machinery at the molecular level (reviewed in Caetano-Anollés et al. 2008, 2012; Caetano-Anollés et al. 2021). Global evolutionary studies of domains defined at different levels of structural abstraction of SCOP (beginning with Caetano-Anollés and Caetano-Anollés 2003) and CATH (Bukhari and Caetano-Anollés 2013) revealed the existence of biphasic patterns of evolution associated with the generation of modules and hierarchy in biology (Caetano-Anollés et al. 2019). A gradual accretion process was also discovered that exhibited a slowdown in domain innovation and four regimes of allometric scaling reflecting a Heaps law and the rise of superkingdoms and viruses (Nasir et al. 2017). Studies also tackled important questions of origin, beginning with metabolism, the ribosome, and the genetic code. An analysis of metabolic enzymes uncovered an origin of metabolism in the purine biosynthetic pathway by gradual replacement of prebiotic pathways followed by patchwork evolution of metabolic networks (reviewed in Caetano-Anollés and Caetano-Anollés 2024). Chronologies of RNA helical structures and domains revealed an origin of the ribosome in the ribosomal ratchet, a process of gradual structural accretion, and co-evolution of RNA and protein structure (Harish and Caetano-Anollés 2012). Similarly, chronologies of dipeptides, domains, and tRNA structures showed that the emergence of codon specificities and amino acid charging was associated with tight coevolution of polypeptides and nucleic acid cofactors (Caetano-Anollés et al. 2013). This coevolution acted as an exacting mechanism favoring flexibility and folding of the emergent proteins. The development of a molecular clock of folds calibrated with biomarkers and geomarkers was particularly remarkable (Wang et al. 2011). As previously anticipated by an exploration of metallome evolution (Dupont et al. 2010), the clock linked the geological record to structural evolution, placing biochemistry within a framework of planetary history. Chronologies also dissected the origin of superkingdoms (Wang et al. 2007) and viruses (Nasir and Caetano-Anollés 2015; Nasir et al. 2017).

## Building evolutionary chronologies

**Fig. 3:**
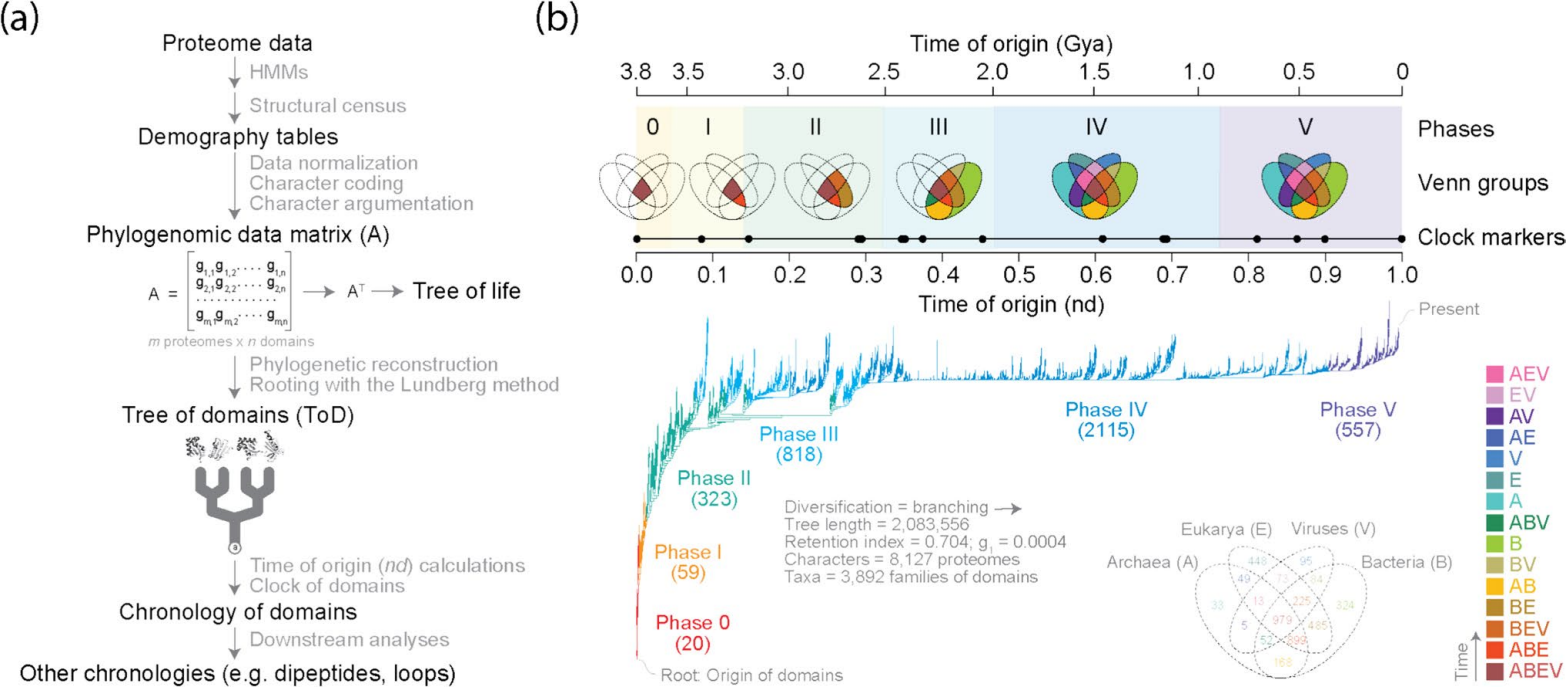
Reconstructing phylogenomic history. **a** A workflow shows the general reconstruction strategy. A census of domains driven by hidden Markov models (HMMs) of structural recognition in proteomes is used to build a demography table and a *nexus*-encoded *m* x *n* phylogenetic data matrix (A) of genomic abundance (g), with *m* representing proteomes and *n* representing domain structures. This matrix and a model of character state change (transformation series) involving ordered multistate Wagner characters are then used to build a tree of proteomes (life) and a tree of domains (ToD) from which to derive chronologies using the “maximum parsimony” criterion and an “heuristic” search strategy. **b** A chronology of domain families built from the ToD shown below (Mughal et al. 2020) illustrates the 6 evolutionary phases of the protein world (shaded periods and colored branches of the tree) and times of origin of molecular clock markers (black circles) that link the biochemical and geological records (Wang et al. 2011). Time of origin was expressed as node distance (*nd*) or as billions of years ago (Gya). Numbers in parentheses represent new families (leaves) appearing in each phase. Phases were defined by Venn diagrams describing the distribution of domain families among Archaea (A), Bacteria (B) and Eukarya (E) and viruses (V). Venn group colors reflect a chronology of Venn group appearance. SCOP concise classification string (*ccs*) labels of clock markers and tree taxa (domains) are not given

Chronologies are arrangements of events in the order of their occurrence and on a fixed temporal scale. In most studies, events represent rare episodes of evolutionary innovation responsible for the first appearance of each domain structure. Chronologies can be derived directly from phylogenomic *trees of domains* (ToDs) reconstructed from genomic censuses of domain structures in proteomes with alignment-free methodologies. We illustrate an experimental strategy with a workflow (Fig. 3a) and a ToD describing the evolution of 3892 SCOP domain families (Fig. 3b). The ToD was reconstructed from domain abundance values in 8127 reference-quality (RefSeq) proteomes representing superkingdoms and viruses. Note that the phylogenetic reconstruction maximizes a fit of phylogenetically informative data to character changes in the branches of trees by traveling through the enormous taxon-delimited tree solution space via branch-swapping operations. Also note that maximum parsimony outperforms maximum likelihood and other methods of phylogenetic inference, especially those involving morphological-type serial homologies (Goloboff et al. 2018). To establish a direction of evolutionary change, ToDs were “rooted” by pulling down a branch containing the ancestor of all taxa (the root node) most parsimoniously using the Lundberg algorithm (Lundberg 1972) and optimizing ancestral-derived homology relations in nested patterns with Weston’s generality criterion (Weston 1994). This rooting approach avoids assumptions introduced by outgroups or molecular clock models.

Rooted ToDs, which are highly unbalanced (i.e., node numbers in left and right subtrees are significantly skewed), allow estimation of an evolutionary order of branching that is indicative of how evolutionarily derived is each leaf of the tree. This order defines a “time of origin” for each domain. A node distance (*nd*) on a relative scale from 0 (oldest) to 1 (youngest) measures the number of nodes from the root node to the leaves and a relative “ancestry” for each domain. Relative times of origin can be made absolute by calibration with a molecular clock of folds, turning *nd* values into billions of years (Gy) of evolution (Wang et al. 2011). Remarkably, the relative positions of domains in timelines generated over two decades of phylogenomic studies have shown consistency, suggesting domain abundance and occurrence carry significant historical signatures and are minimally affected by horizontal gene transfer or sampling biases (Caetano-Anollés et al. 2021).

Chronologies describe a process of accretion and diversification of molecular structures influenced by the spread and abundance of domains across proteomes and by the nesting of homologies in the ToD, which follows Weston’s generality criterion. Domain families that are popular and widely spread (e.g., the TIM barrel structures of metabolism) are found as leaves in deep branches, while families that are less popular and/or confined to specific lineages (e.g., the very popular immunoglobulin domain) are located towards the crown. This outcome is expected. Profile distributions in occurrence and abundance data materialize in a Venn diagram that describes the distribution of domains across the proteomes of Archaea, Bacteria, Eukarya, and viruses (Fig. 3b). However, tracing the 15 Venn groups of the diagram along the chronology consistently shows a sequential appearance of Venn groups, common ancestors, and stem lines of descent defining six evolutionary phases and two universal ancestors: the Last Universal Common Ancestor (LUCA) and the Last Universal Cellular Ancestor (LUCellA). This unexpected outcome has been congruently recovered with different datasets and levels of domain abstraction. Phase 0 embodies a “communal world” populated by domains universally present in cells and viruses (Venn group ABEV). Phase I describes the “rise of viral ancestors” by splitting the ABEV families into those only common to superkingdoms (ABE). Phase II defines the “birth of archaeal ancestors” with novel families (BE and BEV groups) characteristic of an ancestral cellular stem line undergoing reductive evolution. Phase III describes the rise of “diversified bacteria” with about half of Venn groups, all of them involving bacterial families. Finally, phases IV and V introduce the remaining Venn groups, including eukaryote-specific families (E) and virus-specific families (V) typical of domains involved in viral life cycles (e.g., capsids). This detailed analysis highlights the complex evolutionary relationships and temporal patterns of domain structures unfolding across diverse life forms and viruses.

## Tracing the evolution of loop prototypes, the elemental architects of protein structure

Structural chronologies describe the evolutionary accumulation of building blocks (Caetano-Anollés et al. 2008). These elements are “prior molecular states,” intermediate forms (precursors) that often manifest as evolutionarily conserved “living fossils” in present-day protein structures (Caetano-Anollés et al. 2024). Besides domains, proteins harbor smaller elements, including *k*-mers and dipeptides (Pe’er et al. 2004; Choi and Kim 2020; Amangeldina et al. 2024). For example, protein folds embed loop structures (Leszczynski and Rose 1986). These “supersecondary motifs” (*Smotifs*) comprise non-regular (aperiodic) regions that span secondary structures and direct returns of the protein backbone in space (Oliva et al. 1997; Fernandez-Fuentes et al. 2010). Loops play essential structural, functional, and evolutionary roles (Papaleo et al. 2016; Corbella et al. 2023), acting as autonomous evolutionary units (Romero Romero et al. 2016; Aziz et al. 2016; Berezovsky et al. 2017; Heizinger and Merkl 2021). Stand-alone loops, 9–39 residues long, are thought to be remnants of ancient peptides that existed in a primordial RNA-peptide world (Alva et al. 2015). Combinable loops, 25–30 residues long, form active sites and bind cofactors forming “elementary functional loops” (EFLs) (Berezovsky et al. 2000; Goncearenco and Berezovsky 2015). Longer “themes,” 35–200 residue long, are conserved structural building blocks that decrease their reuse with increasing length (Nepomnyachiy et al. 2017). Machine learning (e.g., HMMs) and alignment-dependent techniques (e.g., sequence profiles) have identified and clustered these conserved subdomain fragments. While relationships may not necessarily reflect evolution because of processes of divergence and convergence, the very popular nucleotide-binding EFLs that are glycine-rich (Zheng et al. 2024) and others (Berezovsky et al. 2017) are likely very ancient. Their chronological tracing throughout domain history reveals massive recruitment (Aziz et al. 2016).

**Fig. 4:**
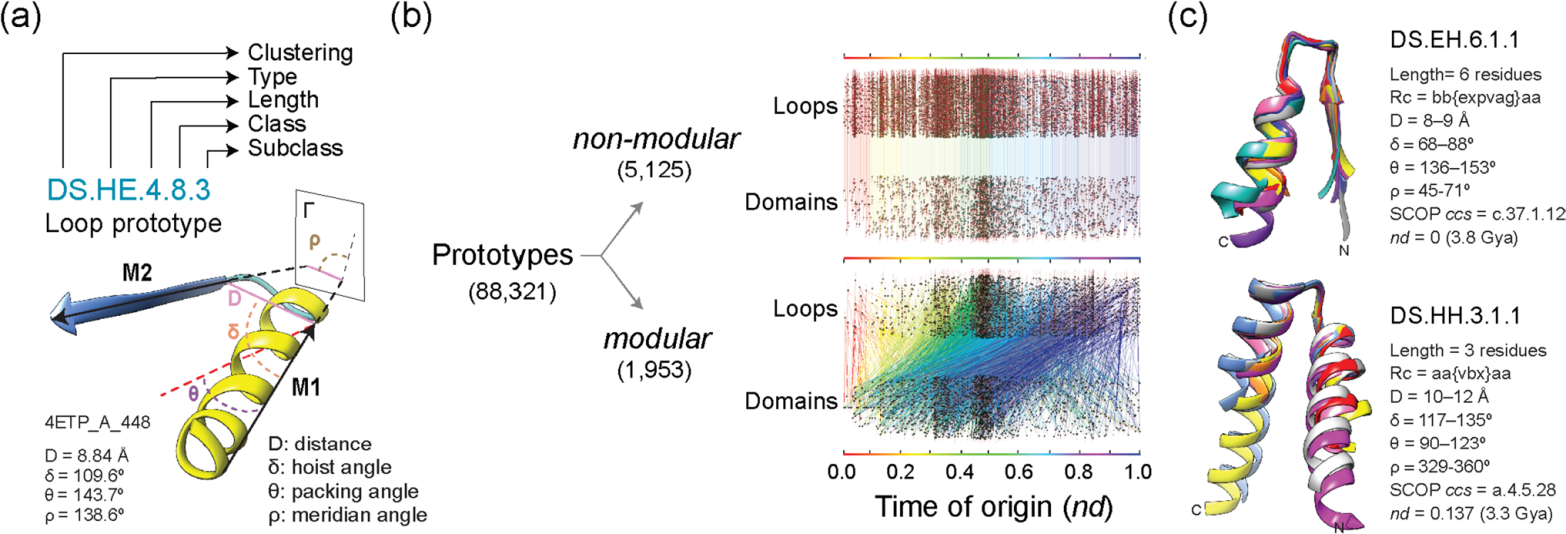
Tracing the evolution of loop prototypes. **a** Loop prototypes in ArchDB are defined by clustering method (DS of DHL), bracing secondary structure of the loop (type), number of residues forming the aperiodic structure (length), conformation given as a Ramachandran consensus (Rc) of φ and ψ backbone dihedral angles (class), and the geometry of the loop measured using four internal coordinates (D, δ, θ, ρ) extracted from the orientation of principal vectors (M1 and M2) of the bracing secondary structures (subclass). The atomic model of loop 4ETP_A_448 illustrates the 4-level classification system. **b** Mapping loop prototypes (extracted by DS clustering of 306,725 ArchDB loops) to domain families allowed to place prototypes along chronologies, assign times of origin (*nd*), and identify prototypes that were either *non-modular* or *modular* (numbers in parentheses) in evolving bipartite networks of prototypes and domains. **c** Aligned families of loop structures represent modular prototypes responsible for the first primordial waves of functional innovation and recruitment. The phosphate-binding prototype DS.EH.6.1.1 holds the P-loop of the ABC transporter ATPase domain-like family. Prototype DS.HH.3.1.1 holds the helix-turn-helix (HTH) motif of the winged helix domain of transcriptional regulators. Data from Aziz et al. (2023)

We used ArchDB (Bonet et al. 2014) to map loop structures to domains and study their evolution (Aziz et al. 2023; Mughal and Caetano-Anollés 2023). ArchDB extracts *Smotifs* from known Protein Data Bank (PDB) structural entries and classifies them into “loop prototypes” based on the bracing secondary structures of the loops and parameters of conformation and geometry (Fig. 4a). The loop prototype library has been considered complete since 2000 despite PDB growth (Fernandez-Fuentes et al. 2010). Mapping prototypes identified with Density Search (DS) clustering along chronologies revealed two types of prototypes, modular and non-modular (Fig. 4b). Modular prototypes were fewer but widely used, while the numerous non-modular prototypes were restricted to specific origins and time periods. These findings align with the earlier observations that a small set of common prototypes encompass most known protein folds. Remarkably, a time series of evolving bipartite networks describing the recruitment of modular loops by domains revealed dense recruitment patterns throughout the timeline (Aziz et al. 2023), especially starting halfway along the evolutionary progression (Fig. 4b). Networks uncovered two major waves of functional innovation centered around the “P-loop” and “winged helix” loop structures (Aziz et al. 2023). The P-loop is embedded in the ancient and widely used Rossmanoid α-β-α layered fold structure crucial for nucleotide triphosphate-binding, and the winged helix motif is embedded in a bundle structure vital for interactions with nucleic acids and proteins in key cellular processes like transcription (Fig. 4c). Both waves recruited cysteine-rich prototypes and three essential SCOP superfamilies (SCOP *ccs*: c.2.1, c.66.1, and b.40.4) necessary for translation and other core biological functions. The centrality of α-β-α layered sandwich, β-barrels, and helical bundle folds was confirmed by studies of metabolic evolution (Caetano-Anollés et al. 2007) and chronologies of early structural domains (Caetano-Anollés et al. 2007, 2012), providing strong evidence of their evolutionary significance.

## The birth of domain structural cores

**Fig. 5:**
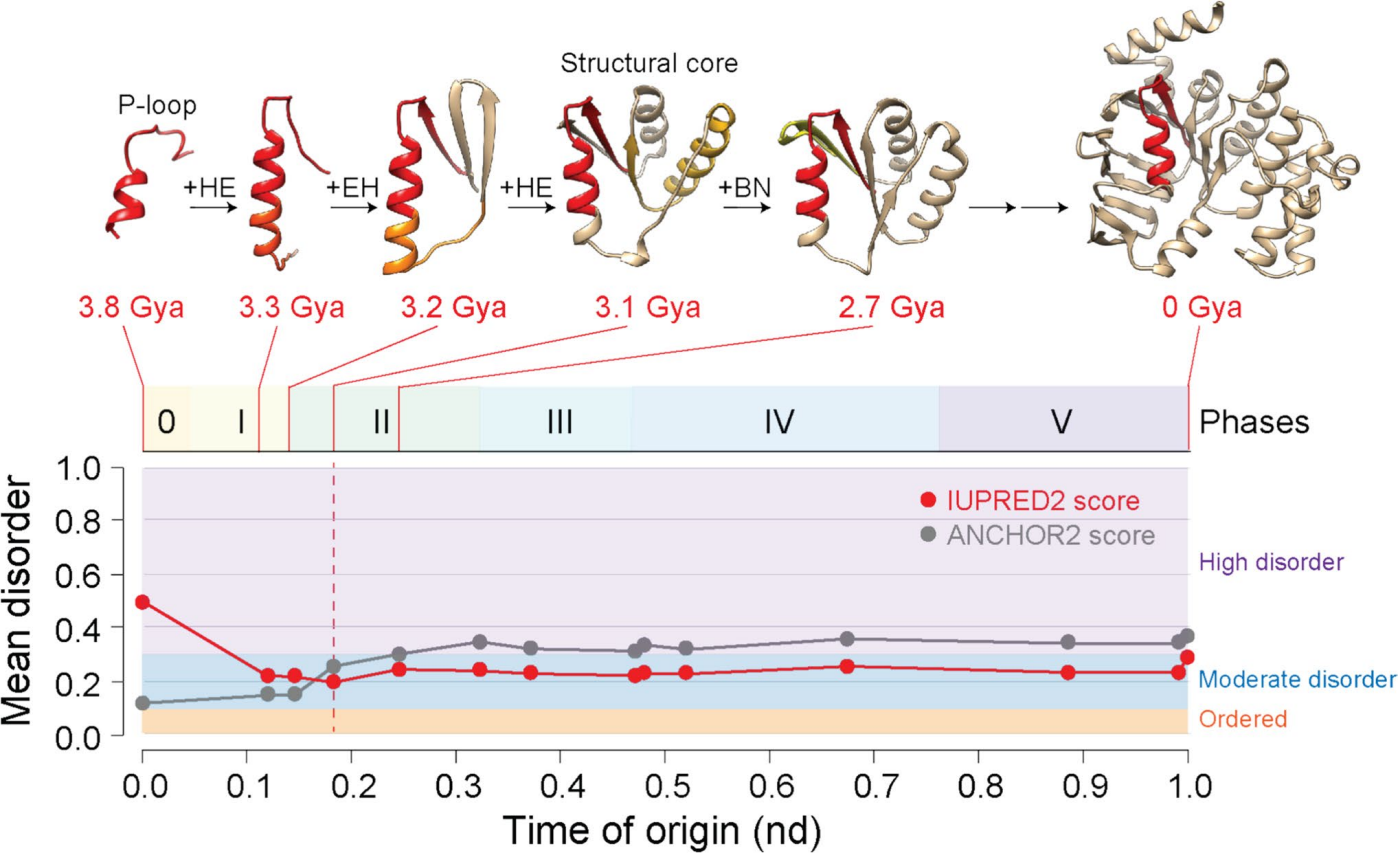
Piecemeal buildup of the oldest domain family, the P-loop containing ATP-binding domain by individual steps of loop accretion. Each step of the time-ordered series of AlphaFold2 modeled structures adds a new loop prototype (identified only with structural types) to the growing structure. In the process, accretion decreases intrinsic disorder measured using UIPred2A (short) scores (Mészáros et al. 2018; Erdős and Dosztányi 2020). Anchor2 scores measure disorder binding. AlphaFold2 models can be downloaded from ModelArchive (https://doi.org/10.5452/ma-gca-proto). Modified from Aziz et al. (2023)

Tracing the origin of loops onto atomic models of domains described the birth and piecemeal buildup of protein structures in evolution. The molecular accretion was effectively modeled at atomic resolution using the deep learning AlphaFold2 ab initio predictive computational tool with high confidence (Aziz et al. 2023). Modeling showed how central cores of domains materialized relatively quickly by accretion of prototypes. Figure 5 describes the formation of the central core of the oldest domain structure, the “ABC transporter ATPase domain-like” family (c.37.1.12). This core, which packs the helical bracing structure of its P-loop against an emergent sheet of three β-strands, unfolded within a period of 0.7 Gy in phase II. In this process, the “high disorder” levels of the founder P-loop (mean disorder of 0.483) decreased to “moderate disorder” levels (0.184–0.278) by subsequent steps of loop accretion. Similar tendencies towards order appeared in the evolution of other domains, linked to variation in the size and structure of the molecule. These models suggested structural reformation as a common phenomenon during accretion, explaining why the high disorder of recruited loop prototypes [mean disorder of 0.626 ± 0.219 (SD) for the 13 prototypes of c.37.1.12] does not maintain when being part of the growing domains.

## Evolution of intrinsic disorder

**Fig. 6:**
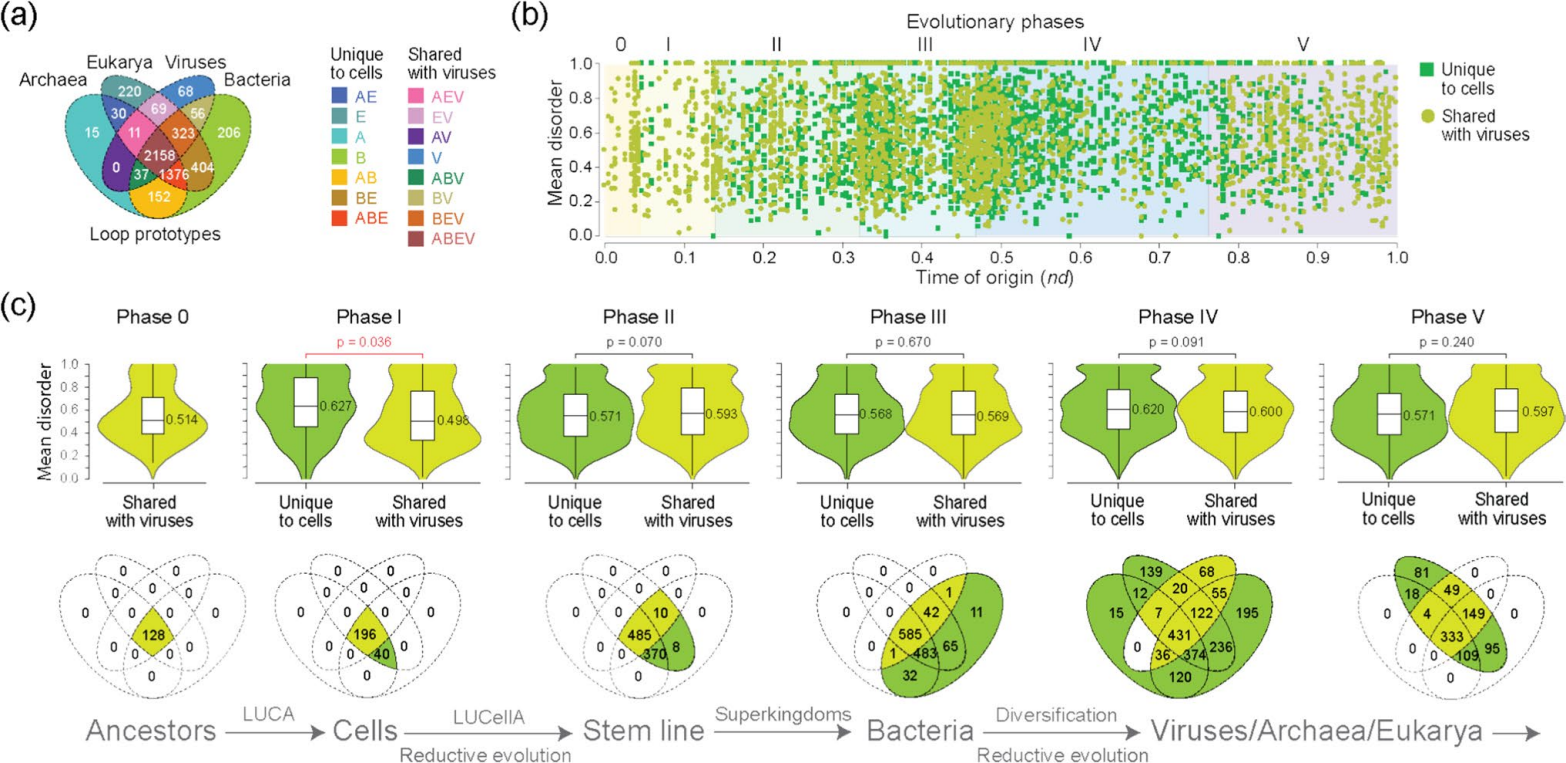
Evolution of intrinsic disorder in protein loops. **a** A four-set Venn diagram describes the distribution of non-modular prototypes in the protein world. **b** Mean disorder scores of non-modular prototypes that are unique to cells or shared with (or unique to) viruses are plotted along the timeline. **c** Violin plots compare the mean disorder scores of prototypes of both groups originating in each of the 6 evolutionary phases. Prototype numbers in Venn groups for each phase are only compatible with the model (timeline) of cellular evolution shown below. Data from Mughal and Caetano-Anollés (2023)

Intrinsically disordered regions that are flexible and lack fixed atomic structure under native conditions are ubiquitous in the proteins of cells and viruses (van der Lee et al. 2014; Oldfield et al. 2019). Their biophysics enables flexibility, binding, and function at the molecular level, which can be explained by a “structure–function continuum” of protein dynamic and functional behavior (Uversky 2016). Little is known however about the evolution of disorder. In order to understand evolutionary patterns intimated by our AlphaFold2 modeling experiments, we first traced mean disorder along chronologies of loop prototypes (Mughal and Caetano-Anollés 2023). We focused on the more numerous non-modular prototypes to avoid dissecting evolutionary recruitment. The distribution of 5125 prototypes in 8127 proteomes identified those that were unique to cells or shared with viruses (Fig. 6a). These groups were then mapped along the chronology (Fig. 6b) to test the impact of cellular evolution on protein disorder (Fig. 6c). Most prototypes in both groups exhibited “high disorder” levels, with values spanning the entire 0.3–1.0 range of mean disorder scores. The widely distributed ABEV and ABE prototypes originated in all 6 and in the last 5 phases of the protein world, respectively, highlighting their continued emergence (Fig. 6c). However, disorder medians were only increased at significant levels (two-tailed Wilcoxon rank sum test; *W* = 3111.5, *z* = 2.05; *p* < 0.05) in the stem line leading to LUCellA in phase I during the rise of primordial viruses. The increase in disorder solidified in subsequent phases for all emerging prototypes and stem lines, showing the evolutionary advantage of high disorder in the makeup of loops.

**Fig. 7:**
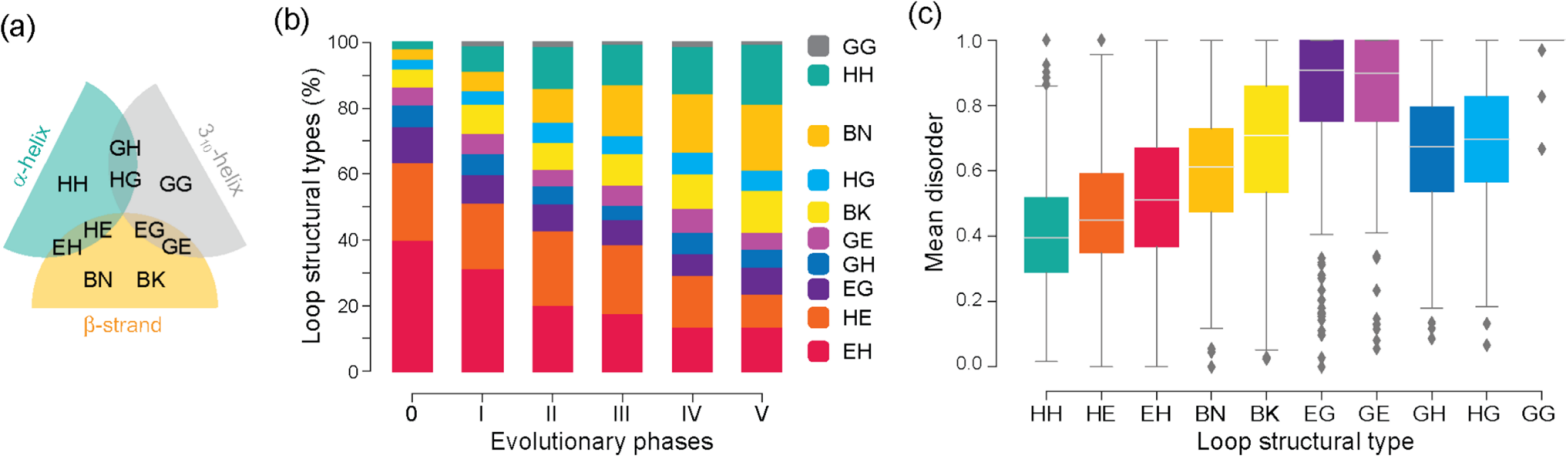
Structural types of loop prototypes and their disorder. **a** Venn diagram of bracing secondary structures (described with DSSP letter codes) of the 10 types. **b** Evolution of types along phases. **c** Distribution of mean disorder scores in types. Data from Mughal and Caetano-Anollés (2023) and Caetano-Anollés et al. (2024)

The lower levels of disorder in prototypes shared with viruses appearing in phases 0 and I can be explained by their structural types (Fig. 7a). The popularity of types with α-helix and β-strand bracing structures (EH and HE) at the beginning of the timeline was offset by a gradual increase of all other types, especially the β-strand and β-strand (BN and BK) motifs (Fig. 7b). The early overrepresentation of the EH and HE types probably stems from the benefits of packing helical structures onto small sheets of β-strands typical of ancient α-β-α layered fold structures (Caetano-Anollés et al. 2012). Remarkably, the EH and HE types are more ordered than other types, with the notable exception of HH (Fig. 7c), explaining how their early overrepresentation would bring mean disorder levels down in early prototypes.

**Fig. 8:**
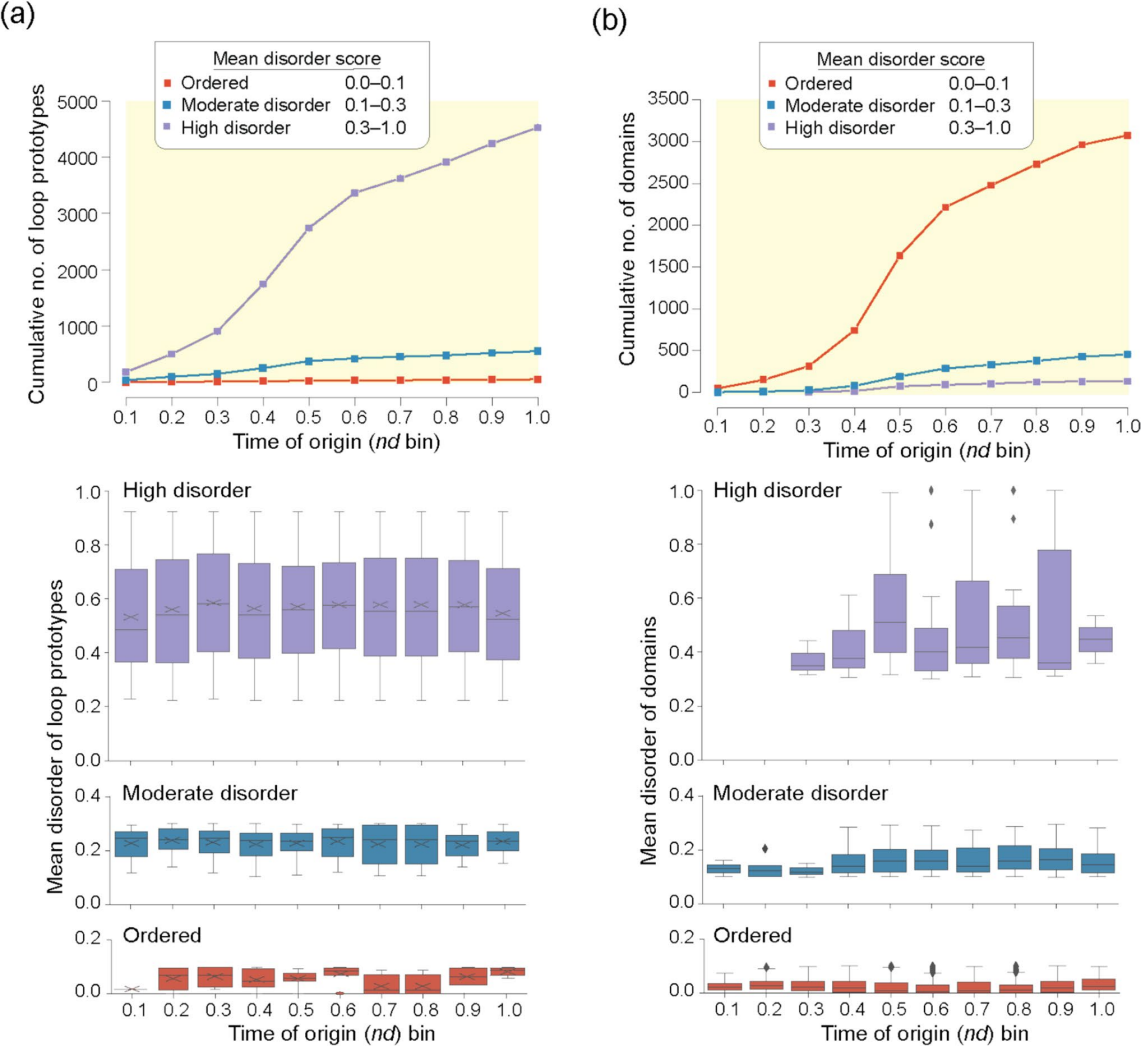
The evolution and intrinsic disorder of loop prototypes (**a**) and domains (**b**). Plots describe the accumulation of structures (top) or the distribution of disorder (bottom) in the “ordered,” “moderate disorder,” and “high disorder” categories that were appearing in the bins of the timeline. Data from Mughal and Caetano-Anollés (2023, 2024)

A more detailed mapping of “ordered,” “moderate disorder,” and “high disorder” prototypes along the chronology visualized as both accumulation plots or as disorder distribution plots confirmed early evolutionary tendencies towards high disorder and late evolutionary development of ordered and moderate disordered prototypes (Fig. 8a). Surprisingly, extending this type of analysis to 3892 domain families in 8127 proteomes (Mughal and Caetano-Anollés 2024) uncovered an opposite evolutionary tendency (Fig. 8b). Most domains were ordered, order appeared early in evolution, and moderate disorder and high disorder were derived. Thus, evolution of disorder depends on the length of the molecules being considered. While the most ancient loops were already highly disordered, an evolutionary push towards disorder along the timeline highlights the central evolutionary role that flexibility has in the structures of building blocks. In contrast, the most ancient domains were significantly ordered, with disorder evolving as a benefit acquired later in evolution.

**Fig. 9:**
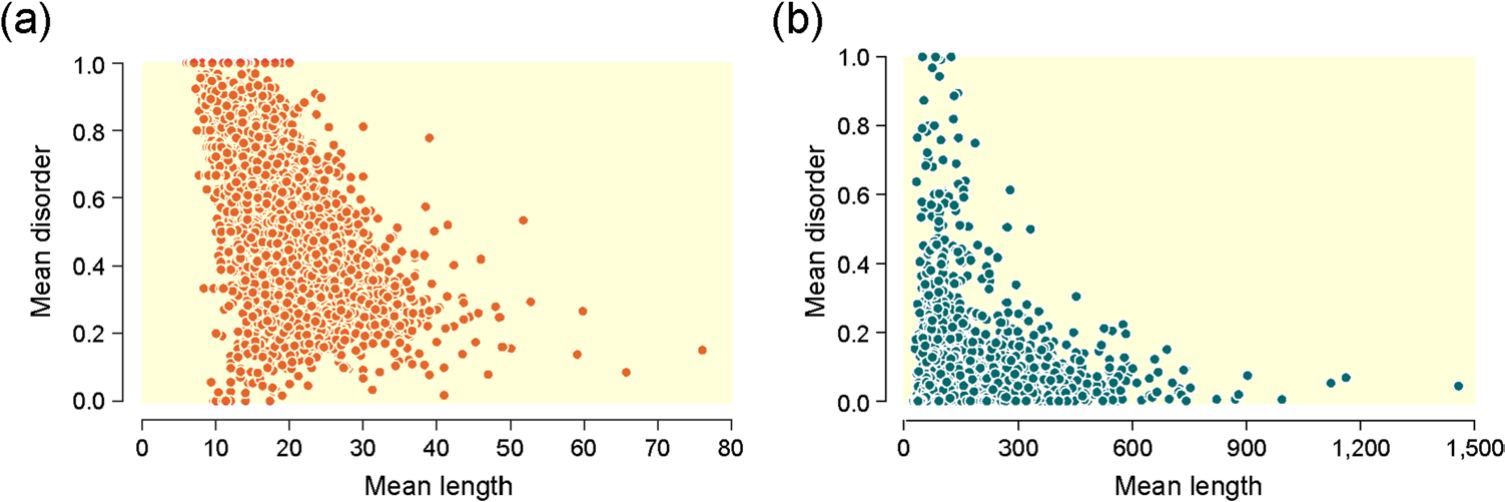
Mean disorder scores of loop prototypes (**a**) and domains (**b**) plotted against mean length show significant correlations. Data from Mughal and Caetano-Anollés (2023, 2024)

Evolutionary tendencies imposed by molecular growth must materialize in patterns that exist in the extant world. Indeed, plotting mean disorder against mean sequence length of both loops and domains showed significant negative correlations (Spearman’s rho, *p*-values < 0.001), highlighting the central role that molecular size plays in protein evolution (Fig. 9). Note that a remarkable biophysical link exists between protein size and both folding and stability of the native state (De Sancho et al. 2009), allowing the prediction of folding-unfolding rates using just 10-bits of information in size and structural class (De Sancho and Muñoz 2011). Under the folding speed limit of ~ 1 μs established by measuring conformational motions with ultrafast kinetic techniques, folding free energy barriers estimated from rates become entropic bottlenecks that scale with size (Campos et al. 2020). For microsecond folders, including fold archetypes and domains, folding either crosses small barriers or simply occurs downhill. For polypeptides of less than 50 residues, such as many loop prototypes, folding free energy barriers are expected to be marginal (< 12 kJ/mol). Unexpectedly, folding-upon-binding of disordered regions appears to function as a conformational rheostat (Campos et al. 2020). This rheostat can fold prior to binding via conformational selection or fold downhill after binding through induced fit, prompting folding-unfolding conformational transitions. The evolutionary link between size and folding speed, traced in domain chronologies (Debès et al. 2013), is therefore complex and partly responsible for the disorder-size scaling patterns of Fig. 9.

## Concluding remarks

Historical chronologies inspired and driven by phylogenomic data demonstrate the role of protein loops as stepping stones toward domain structure (Aziz et al. 2016; 2023). Loop recruitment seems to be a continuous evolutionary process that modulates disorder in a growth-dependent and loop-dependent manner (Mughal and Caetano-Anollés 2023, 2024; Caetano-Anollés et al. 2024). Evolutionary trends resulted in larger proteins being more structured than smaller and more disordered proteins. These trends reflect the diverse constraints imposed by the expanding functional roles of disordered regions, particularly in eukaryotes and viruses.

While loops are embedded in domains (Berezovsky et al. 2000, 2017), they were once self-standing units (Caetano-Anollés et al. 2024). In fact, loops are still present in non-ribosomal biologically active peptides (Flissi et al. 2020) or emergent disordered molecules responsible for de novo gene creation (Wilson et al. 2017; Kosinski et al. 2022). Peptide disorder deviates from a “random coil” (unfolded state) model, emphasizing the importance of conformation dynamics and helping understand how residue and size-dependence are functions of internal or external parameters (Schweitzer-Stenner 2023). For example, modulation of promiscuous binding via “handles” (e.g., phosphate moieties) may have been a primordial feature in emergence of functional protein structure (Noor et al. 2022). Here, uniform binding mechanisms that do not require discrimination between substrates, products, and transition states could have provided fitness advantages to metabolic reactions, in particular, by channeling interactions towards desired products through negative catalysis. Remarkably, phylogenomic analysis of Gene Ontology (GO) terms of molecular function defined at levels 2 and 3 of classification shows “binding” terms appear immediately before “catalytic activity” terms in evolutionary chronologies (Koç and Caetano-Anollés 2017), supporting the uniform binding model and its primordial role during protein emergence.

Evolutionarily, peptides and proteins frequently favor disorder for flexibility, allowing adaptation to diverse functional roles, such as binding to multiple partners, undergoing conformational changes, or engaging in real-time disorder-to-order transitions (Ahrens et al. 2017). Bellay et al. (2011) used comparative genomic and genetic interaction criteria to classify the structural and functional roles of disorder into three distinct categories: *flexible disorder* (conserved disorder with rapidly evolving sequences), which is associated with signaling pathways and multifunctionality; *constrained disorder* (conserved disorder with highly conserved sequences), primarily involved in nucleic acid binding and chaperone interactions; and *nonconserved disorder*, which lacks specific functional hallmarks. The evolution of intrinsic disorder however encompasses a much larger context that is independent of thresholds, involves functional order–disorder interactions, and contributes appropriately to natural molecular variation (Ahrens et al. 2017). Molecular recognition features (MoRFs) for example are relatively short (10–70 residues) and widespread elements in disordered regions that transition to order upon binding to other proteins or nucleic acids (Mohan et al. 2006; Yan et al. 2016). These transitions are often evolutionarily conserved and result in adoption of secondary structures (helical, strand, irregular, or mixtures of them) important for a range of molecular functions. Disorder also modulates remote regulatory effects of binding on functional molecular sites (Tee et al. 2020), which are central to the engineering and design of controllable allosteric signaling (Tee et al. 2022). The observed order-to-disorder transition in evolution of loops may be already reflected at single residue level in the regulation of allosteric signaling that gauges structural stability and conformational change (Tee et al. 2020). Mapping MoRFs, allosteric residues, and binding regions to loops and domains along chronologies could help dissect their evolutionary contributions to molecular flexibility, conformation, and function throughout protein history.

We end by noting that small and large proteins evolve well-defined structures to stabilize specific functions. However, even large multi-functional proteins incorporate disordered regions, especially at the termini or between structured domains. These disordered regions play a fundamental role in maintaining the balance between flexibility and specificity, enabling adaptive responses to functional or environmental change, and enhancing the functional diversity of proteins (Ahrens et al. 2017). In protein evolution, the interplay between order and disorder appears to govern the dynamics of folding, partitioning, and aggregation. Understanding this balance would provide insight into the evolution of protein functionality, addressing disorders associated with protein misfolding and aggregation, such as neurodegenerative diseases.

## Data Availability

No datasets were generated during the current study.
